# Anesthetic management of a pediatric patient with Electron Transfer Flavoprotein Dehydrogenase deficiency (ETFDH) and acute appendicitis: case report and review of the literature

**DOI:** 10.1186/s12871-017-0400-9

**Published:** 2017-08-29

**Authors:** Emmanuel Lilitsis, Elisavet Astyrakaki, Evaggelos Blevrakis, Sofia Xenaki, George Chalkiadakis, Emmanuel Chrysos

**Affiliations:** 1 Department of Anesthesiology, University Hospital of Crete, Herakllion, Greece; 2 Department of Pediatric Surgery, University Hospital of Crete, 71110 Herakllion, Greece

**Keywords:** Electron Transfer Flavoprotein Dehydrogenase, Deficiency, Pharmacological anesthetic agents

## Abstract

**Background:**

Mitochondria are the energy producing organelles practically in every human cell except erythrocytes. Indeed mitochondria are widespread in high energy requiring organs like brain, heart and muscles. Currently there are no clinical trials supporting with clear evidence which is the most suitable surgical or anesthetic management of a patient with known mitochondrial disease presenting with surgical disorders. This condition poses possible hazardous problems to the medical attention of those patients.

**Case presentation:**

A case of an 8 year old child with known Electron Transfer Flavoprotein Dehydrogenase deficiency (ETFDH deficiency) requiring surgery for acute appendicitis is presented. Our approach for anesthesia revealed a combination of fentanyl, low dose propofol and nitrous oxide.

**Conclusion:**

The choice of the safest pharmacological anesthetic agents for patients with ETFDH deficiency is challenging given that most of the general anesthetic medications have multiple effects on mitochondria, fatty acids metabolism and striated muscles. Anesthetists are expected to individualize anesthetic care for the patient based on current publications for similar cases, medical history and knowledge of pharmacology and physiology.

## Background

Mitochondria are the energy producing organelles practically in every human cell except erythrocytes. Indeed mitochondria are widespread in high energy requiring organs like brain, heart and muscles. Almost every ATP producing metabolic pathway except glycolysis takes place in mitochondria. Krebs cycle, oxidate phosphorylation and fatty acid oxidation are the main pathways for the generation of triphosphate adenosine (ATP). Mitochondrial disorders represent a group of enzymatic defects in respiratory chain involved in the formation of ATP [1]. There are over 1000 enzymatic procedures known to participate in oxidate phosphorylation so not strangely the incidence of respiratory chain disorders is considered to be 1 per 4000 live births affecting clinically mainly nervous and muscular system including the striated heart muscle [2].

A case of an 8 year old child with known Electron Transfer Flavoprotein Dehydrogenase deficiency (ETFDH deficiency) requiring surgery for acute appendicitis is presented. Fatty acids are the main energy supply for muscles both at rest and during aerobic exercise while ketones supply brain cells during periods of fasting when glucose levels drop. Heart muscle also rely on fatty acids for energy source. Fatty acids are metabolized in mitochondria via beta-oxidation to acetyl-CoA. Multiple acyl-CoA dehydrogenation deficiency (MADD) are caused by mutations in the gene encoding the electron transfer flavoprotein dehydrogenase, an enzyme in respiratory chain, resulting in abnormal fatty acid, choline and amino acid metabolism [3]. Clinically patients develop encephalopathy, muscle weakness, myopathy, rhabdomyolysis, cardiomyopathy and are extremely susceptible to hypoglycemia. Patients solely depend on glucose for ATP since the beta-oxidation pathway is dysfunctional or insufficient to maintain energy delivery to sensitive organs during fasting [4].

Currently there are no clinical trials supporting with clear evidence the most suitable surgical or anesthetic management of a patient with known mitochondrial disease presenting with surgical disorders. This condition poses possible hazardous problems to the medical attention of those patients. Doctors often extrapolate results and therapeutic approaches from similar conditions for the therapeutic management of these patients. Additionally, even though there are few published case data for the anesthetic management of patients with mitochondrial defects and myopathies with no reported adverse events, this does not necessarily proves the superiority regarding safety of one anesthetic plan over another since most reports are case reports or case series leading the doctors to bias [5].

## Case presentation

An 8 year girl was admitted to the pediatric surgical department with right lower abdominal pain and multiple episodes of vomiting lasting 24 h. Abdominal ultrasound revealed a case of acute appendicitis. Blood count exams were abnormal with elevated white blood cells (15.4 K/μLt) but with normal hematocrit and platelets. Biochemistry exams revealed normal glucose and renal function test but elevated CK (412 U/Lt), SGOP (78 U/Lt), SGPT (53 U/Lt) with normal γ-gt, ALP, billirubin and electrolytes. Parents informed the surgeons that their daughter was diagnosed in infancy with ETFDH deficiency after suffering multiple episodes of hypoglycemia and persistent elevated CK levels on biochemistry. Muscle biopsy confirmed diagnosis and DNA test revealed de novo gene mutation since none of the parents was carrier of the gene mutation. Clinically apart from the abdominal pain the child had normal muscle strength, gait and neurologic examination matched a child of her age. Speech, mental and intellectual level probably above the range according to her age. Indeed she was a piano player and was about to give a piano concert next month. Weight was 30 kg which was normal for her height and age. She had no history of any neurologic pathology including epilepsy. Cardiac ultrasound and ECG performed annually were normal and parents informed that she had a completely normal life with no restrictions at all except the absolute necessity for often oral intake of glucose containing regimens to avoid hypoglycemia. As for prior surgeries, the parents reported a muscle biopsy at a different hospital while the patient was an infant and they had been informed that no adverse events occurred. The child was not under regular medication and no allergies were reported.

A 22 G cannula was inserted and infusion of D/W 10% started according to blood glucose. A venous blood sample was obtained for lactate and pH assessment (pH 7,37, PO2 29 mmHg, PCO2 49 mmHg, HCO3- 25 meq/lt, base excess −1, lactate 8 mg/dl) which were normal. Blood pressure was 112/68 mmHg, heart rate slightly elevated 126/min (possibly due to dehydration) but afebrile (36,7 C). She was normal on cardiac and lung auscultation and airway assessment obtained a possible easy intubation scenario. Proper antibiotic therapy was started with second generation cefalosporin and metronidazole and the child was prepared for surgery.

The anesthesia breathing circuits were replaced with new including the soda lime and the machine was flushed with 100% O2 for five minutes to assure the absolute lack of halogenated gases since previous surgery was conducted with sevoflurane. The patient received no premedication and was transferred to the operation room with a D/W 10% infusion. She was connected to the monitor and pre oxygenation with 100% O2 was started. Vital signs were normal. Induction of anesthesia was conducted with 80 micrograms of fentanyl, 90 mg of propofol and paralysis with 18 mg (0,6 mg/kg) of rocuronium. Intubation was easy and successful with a Murphy tube No 6 with a cuff properly inflated. She was connected to the anesthesia machine on volume control ventilation with Vt 220, respiratory rate 22, PEEP (positive end expiratory pressure) 5. Anesthesia then was maintained with propofol at 0,08 mg/kg/min (144 mg/h or 4,8 mg/kg/h) along with N2O/O2 mix at 55%/45% with a MAC recorded 0,5. Blood pressure after the induction was 95/58 with a heart rate 110/min. Capnography was normal with a EtCO2 34 mmHg and core temperature monitoring was stable with a reading around 36,6-36,9 C degrees during surgery.

After administration of 50 mcg of fentanyl surgery was initiated. Paracetamol 450 mg, ondansetron 4 mg and morphine 1 mg were gradually given. Duration of surgery was 40 min and the patient was completely stable from all aspects. During surgery a venous blood sample was taken and was normal (pH 7,41, PO2 48 mmHg, PCO2 43 mmHg, HCO3- 24 meq/lt, base excess −1, lactate 8 mg/dl, glucose 107 mg/dl). A second venous cannula was inserted for the infusion of 0,9% N/S (avoiding Ringer lactate solution) to replace pre surgical losses due to vomiting. Surgery was uneventful and macroscopically the appendix was inflammatory and removed successfully. Heart rate was 96/min after fluid bolus, EtCO2 remained around 35 mmHg and temperature was stable. Finally sugammadex 60 mg (2 mg/kg) was administered to the patient and she was extubated uneventfully 10 min later. At recovery room the child remained completely stable with no complain of pain or nausea. One hour later she was transferred to the surgical ward. During the first day the patient was monitored for glucose, vital signs along with temperature and a new ECG. Four days later the patient was discharged healthy.

## Discussion and conclusions

ETFDH is a congenital mitochondrial disorder mainly of fatty acids metabolism leading to myopathy, rhabdomyolysis and susceptibility to hypoglycemia. The disease may also be exacerbated during fasting, physical exercise, febrile illness, infection and surgery [6]. A major dilemma appeared after the anesthesia team was informed about the medical history of the patient. The case was acute with no time to postpone or perform more analytical tests. The use of halogenated factors on patients with myopathy could possibly trigger malignant hyperthermia, a fatal but considerably rare complication during general anesthesia due to succinylcholine and volatile halogenated anesthetic agents [7]. On the other hand it is also well known than propofol infusion syndrome (PRIS) is not anymore a rare diagnosis in the critically ill (albeit more often) but there are numerous cases of patients developing this potentially lethal syndrome especially when disorders in fatty acids metabolism and mitochondrial defects co exist [8, 9].

Since there are no guidelines and there are few published data regarding management of such group of patients, medical decisions had to be made out of patient’s symptomatology and limited studies on similar case reports about the effect of known anesthetic agents on the pathophysiology of mitochondrial deficiencies and coexisting myopathies [1, 10–13].

Perioperative fasting shifts energy production to fatty acids through beta oxidation. ETFDH deficiency patients cannot utilize fat as energy so they are extremely susceptible to hypoglycemia as soon as glucose levels drop. D/W 10% was continuously infused with tight glucose control to avoid hypo or hyperglycemia [14]. High glucose levels can also increase lactate acid through increased oxidation [15]. Lactate solutions were withheld due to increased lactate load and possible worsening of metabolic acidosis. Instead N/S 0,9% was used to replace volume deficit [16].

Most of the anesthetic agents have a negative impact on mitochondrial function. Propofol and halogenated agents like sevoflurane, the most commonly used agents, depress mitochondrial function and possibly in multiple ways. Studies support that sevoflurane suppress Complex I, a NADH dehydrogenase (oxidate phosphorylation element), and children with mitochondrial pathology exhibit increased sensitivity [2, 10, 17]. The risk of malignant hyperthermia (MH) development in myopathic patients is not widely accepted. MH is triggered by halogenated agents and succinylcholine and is due to ryanodine receptor mutation leading to abnormal calcium homeostasis. There is clear evidence about MH association and central core disease, mini core disease and King Denborough syndrome, but also and in other types of myopathy [18]. Although there is no unanimous agreement about absolute safety of those anesthetic agents and in many cases induction or even maintaining was managed with sevoflurane, it is probably preferred to avoid triggering factors to those patients [3, 7, 19–21]. Indeed statistically the risk of developing MH or rhabdomyolysis during exposure to a volatile anesthetic agent for patients with myopathy is around 1,09%, considerably high for a potential lethal complication [22]. On the other hand, avoiding bias, numerous studies support the safety of inhalation anesthesia [1]. Muhammad et al. during a national survey in US recorded the use of inhalation anesthesia both for induction and maintenance of anesthesia without adverse outcome, indeed most anesthesiologist preferred inhaled agents in comparison to intravenous [23]. Again medical personnel should be aware of feedback bias while reviewing published data [5].

Our anesthesia team decided to avoid the use of halogenated anesthetic agents. Induction of anesthesia was performed with fentanyl and propofol while maintenance with fentanyl, low dose propofol and N2O. Nitrous oxide has been used in cases with myopathy in combination with propofol and is expected to be safe as an complementary anesthetic agent [12–14]. Propofol on the other hand has multiple sites of action on mitochondria like inhibition of beta oxidation, inhibition of oxidative phosphorylation Complex I, II and IV and inhibition of long chain fatty acids transport in mitochondria [24, 25]. Proposed risk factors to PRIS are increased doses (>4 mg/kg/h) for >48 h, critical illness, concomitant steroid use, high fat diet and inborn errors of mitochondrial fatty acid oxidation [9]. We believe our patient had a very low risk for PRIS since the dose that used was low (4,8 mg/kg/h) and for a minimal duration of 40 min. Several studies in patients with myopathies and mitochondrial defects support this hypothesis [12, 14, 23]. On the contrary Farag et al. suggested that children with mitochondrial pathology are prone to develop metabolic acidosis, myocardial failure and need for ICU admission even after short periods of propofol infusion. Our patient was hemodynamically stable and acid base status was assessed pre and perioperatively with no acidosis detected.

Finally, regarding opioids, there are no data about any negative effect of fentanyl on myopathies or mitochondrial defects, except morphine which is found to have some effect on mitochondrial membrane of doubtful clinical importance [23]. We used 0,6 mg/kg of rocuronium as a neuromuscular blocking agent (NMBA). There are reports for increased sensitivity to rocuronium and especially with patients with myopathy who may require lower dose yet it is the drug of choice for rapid sequence intubation in patients with contraindication to succinylcholine. Reversal with sugammadex is acceptable at a maximum dose 2 mg/kg [13, 23]. Unfortunately no pediatric neuromuscular monitoring was available but reversal on block was clinically obvious taking into account that rocuronium was used in low dose.

The choice of the safest pharmacological anesthetic agents for patients with ETFDH deficiency is challenging given that most of the general anesthetic medications have multiple effects on mitochondria, fatty acids metabolism and striated muscles. Our approach with combination of fentanyl, low dose propofol and nitrous oxide proved to be safe for this routine operation with no adverse outcomes. Anesthetists are expected to individualize anesthetic care for the patient based on current publications for similar cases, medical history and knowledge of pharmacology and physiology.

## Abbreviations

ATP: Triphosphate adenosine
ETFDH: Electron Transfer Flavoprotein Dehydrogenase deficiency
MADD: Multiple acyl-CoA dehydrogenation deficiency
MH: Malignant hyperthermia
NO2: Nitrous oxide
PRIS: Propofol infusion syndrome
